# Crystallographic Observation of pH-Induced Conformational Changes in the *Amyelois transitella* Pheromone-Binding Protein AtraPBP1

**DOI:** 10.1371/journal.pone.0053840

**Published:** 2013-02-13

**Authors:** Eric di Luccio, Yuko Ishida, Walter S. Leal, David K. Wilson

**Affiliations:** 1 Department of Molecular and Cellular Biology, University of California Davis, Davis, California, United States of America; 2 Department of Entomology, University of California Davis, Davis, California, United States of America; INRA-UPMC, France

## Abstract

The navel orangeworm, *Amyelois transitella* is a major agricultural pest causing large losses in a variety of tree crops. Control of this insect pest may be achieved by interfering with olfactory pathways to block detection of female-produced sex pheromones and consequently, disrupt mating. The first component of this pathway is the pheromone-binding protein AtraPBP1, which recognizes the pheromone and presents it to the odorant receptor housed in a sensory neuron of the male antennae. Release of the ligand depends on a pH-induced conformational change associated with the acidity of the membrane surface. To characterize this conformational change and to understand how pheromones bind, we have determined the high resolution crystal structures of AtraPBP1 in complex with two main constituents of the sex pheromone, i.e., (11*Z*,13*Z*)-hexadecadienal and (11*Z*,13*Z*)-hexadecadienol. Comparison with the structure of the unliganded form demonstrates a large ∼90° movement of the C-terminal helix which is observed in other pheromone- or odorant-binding proteins accompanied by an unpredicted 37° displacement of the N-terminal helix. Molecular dynamic trajectories suggest that the conformational change of the α1 helix facilitates the movement of the C-terminal helix.

## Introduction

Larvae from the navel orangeworm, *Amyelois transitella* (Walker) infest nuts such as almonds, pistachios and walnuts leading to $50 million of crop damage annually. Insecticides are not particularly effective and can affect beneficial insects so alternative control methods are needed. An environmentally preferable strategy, which can more specifically target a single species, is to apply low concentrations of these pheromones or related compounds that interfere with the insects' ability to find mates in the field. This approach has been used in other species of Lepidoptera and is a proven method of pest control once pheromones have been identified [1]. Similarly, attempts to attract or control *A. transitella* using one or more components of the insect's pheromone mixture have shown positive results [2], [3]. A more authentic synthetic pheromone mixture is more bioactive yet contains unstable compounds which suggest a need for stable pheromone analogs to be developed.

Understanding the structural basis of pheromone detection and elucidating the mechanisms of action of olfactory proteins will aid in the development of novel and stable mimics of one or more of the attractants. These pathways are mediated in part by pheromone-binding proteins (PBPs), which are believe to be important in solubilizing the hydrophobic ligands and serve as an initial transporter that present odorants to a receptor in the membrane to elicit an olfactory response. Some PBPs bind pheromones with high affinity at the relatively high ambient pH present in the sensillar lymph and release them in the lower pH and cationic conditions, which are thought to exist near the membrane [4]. Structural studies of classical PBPs and related odorant-binding proteins (OBPs) have correlated this with a variety of conformational changes, most of which affecting the protein's C-terminal sequence [5]–[9] however a pH-induced domain swap has also been observed [10]. On the other hand, models of cationic (e.g. potassium) concentrations and pH as a function of distance from the membrane have been used to guide titration studies of two PBPs from the gypsy moth *Lymantria dispar*. Experimentally co-varying potassium and pH to simulate predicted concentrations near the membrane yield no change in the dissociation constant [11].

**Figure 1:**
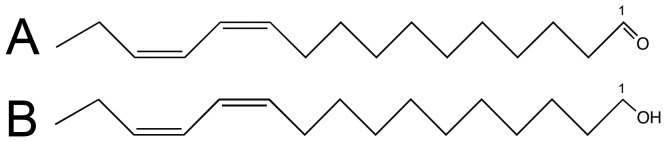
Chemical structures of the major constituents of the *A. transitella* sex pheromone blend (A) the major constituent (11*Z*,13*Z*)-hexadecadienal (*Z*11*Z*13-16Ald) and (B) the secondary constituent, (11*Z*,13*Z*)- hexadecadienol (Z11Z13-16OH).

A primary pheromone-binding protein (PBP) from *A. transitella*, (AtraPBP1) has been identified based on its expression profile in male antennae and its biochemical properties [12]. Xu et al. have determined the unliganded NMR structure of this protein in acidic conditions (pH 4.5), which reduce the affinity for the pheromone [13]. A further study suggests that there is a conformational change associated with the C-terminal region of AtraPBP1 forming an α-helix at this low pH which competes with pheromone binding [8]. The exact nature of this conformational change and the molecular determinants of the discrimination of the sex pheromones blend by the protein are still unknown, however. To answer these questions and to pave the way for the design of a stable synthetic sex pheromone useful as a mating disruptor, we solved the high-resolution crystal structures at high pH of the liganded AtraPBP1 complexed separately with the major constituent of the sex pheromone of *A. transitella*, (11*Z*,13*Z*)-hexadecadienal (*Z*11*Z*13-16Ald) and the secondary constituent, (11*Z*,13*Z*)-hexadecadienol (*Z*11*Z*13-16OH) (Fig. 1).

## Materials and Methods

### Crystal preparation and structure determination

A pET22-b(+) vector containing DNA encoding mature AtraPBP1 was used to transform BL21 (DE3) competent cells (EMD Chemicals, Novagen, Gibbstown, NJ). The transformant was used to inoculate LB medium containing carbenicilin and cells were cultured at 200 rpm at 28°C overnight. After IPTG induction for 3 hours, the recombinant protein was extracted from harvested cells using a freeze-thaw procedure. The recombinant AtraPBP1 was purified by a combination of ion-exchange chromatography and gel-filtration as described previously [4]. Purity of the protein was monitored by HPLC-electrospray ionization-mass spectrometry (LCMS-2010; Shimadzu USA, Colombia, MD).

Purified AtraPBP1 was crystallized at room temperature by the hanging drop vapor diffusion method. Drops composed of 1 µl protein solution (30 mg/ml) and 1 µl of the precipitant solution were suspended over a reservoir containing the precipitant solution (1.6 M sodium citrate pH 6.5). Crystals used in data collection were transferred into Paratone-N oil and flash-cooled in a stream of liquid nitrogen at 110 K. Data sets were taken at SSRL beamline 9–2 and reduced using HKL2000 v 1.98.4 [14]. All AtraPBP1 cocrystals were obtained using stock protein containing either (11*Z*,13*Z*)-hexadecadienal or (11*Z*,13*Z*)-hexadecadienol using a 20-fold molar excess of pheromone. Both crystal forms belonged to the space group *P*6_5_ with one molecule per asymmetric unit and unit cell dimensions of a = b = 57.46 Å, c = 93.27 Å (*Z*11*Z*13-16Ald complex); a = b = 57.53 Å, c = 93.501 Å (*Z*11*Z*13-16OH complex). The Matthews coefficient Vm is 2.76 A**3/Da with a solvent content of 55.5%.

The initial phasing for the AtraPBP1-*Z*11*Z*13-16Ald complex structure came from a molecular replacement solution using PHASER and the *Bombyx mori* pheromone-binding protein structure (Protein Data Bank accession 1DQE) as a search object [15], [16]. The refined AtraPBP1-*Z*11*Z*13-16Ald structure was used to phase the dataset collected from the AtraPBP1-*Z*11*Z*13-16OH complex. Several rounds of crystallographic refinement and manual refitting using the programs COOT and Refmac5 resulted in the final models of both *Z*11*Z*13-16Ald and *Z*11*Z*13-16OH AtraPBP1 complexes (Table 1) [17], [18]. High-resolution refinement at 1.14 Å of holo-*Z*11*Z*13-16Ald AtraPBP1 was performed with hydrogens added in the riding positions along with an anisotropic B-factor refinement. Water molecules were automatically picked in COOT v0.5 and manually checked for appropriate hydrogen-bonding and electron density agreement [17], [19].

**Table 1:** Data collection and final refinement statistics. Values in parentheses are for the highest resolution shell.

Data Collection		
PDB code	4INX	4INW
Ligand	Z11Z13-16OH	Z11Z13-16Ald
Wavelength (Å)	1.2000	1.1000
Resolution range (Å)	50–1.85 (1.92–1.85)	50–1.14 (1.18–1.14)
Unique observations	15,052	63,309
Total observations	63,875	456,474
Completeness (%)	98.3 (91.8)	97.2 (91.4)
Space group	*P*6_5_	*P*6_5_
R_sym_	0.040 (0.113)	0.058 (0.475)
<I/σ(I)>	28.4 (10.1)	29.3 (2.6)
**Refinement**		
Resolution range (Å)	34.10–1.85	28.7–1.14
Reflections used	14,023	55,453
R_cryst_ (%)	16.8	16.0
R_free_ (%)	21.4	18.3
# of protein, non-hydrogen atoms	1,286	1,339
# of non-protein atoms	177	208
rms bond length (Å)	0.022	0.022
rms bond angles (°)	1.87	1.80
Average main chain B values (Å^2^)	13.01	18.18
Average ligand B values (Å^2^)	30.33	33.67

### Modeling and dynamics

The coordinates of Z11*Z*13-16Ald bound AtraPBP1 were subjected to 25000 steps (25 ps) of torsional molecular dynamics (MD) using CNS V1.2 to model in vacuum the motion of the helices α1 and α7 [20]. After the addition of all the hydrogens, all the atoms were harmonically restrained (weight k = 1 kcal/mole/Å^2^) except for the residues 1 to 15 and 129 to 140 corresponding to the helices α1 and α7. The MD trajectories were analyzed with VMD v1.8.7. and rendered in PyMOL v1.2 [21], [22]. The access channel to the inner hydrophobic pocket appeared at 12.5 ps (12500 steps) and its geometry remains unchanged until the end of the MD simulation at 25 ps (25000 steps). The various MD models at t = 5 ps, t = 10 ps, t = 12.5 ps were energy minimized with spatial restraints (CNS V1.2) and manually checked for ideal stereochemistry using Coot [17], [20]. The Hingefind algorithm was used with VMD 1.8.6 to estimate the overall rotation angle of the helices α1 and α7 [21], [23]. Solvent accessibility calculations were performed using Areaimol (CCP4), PISA and CNS.

Structural overlays were done using the programs SUPERPOSE and LSQKAB (CCP4 V.6.0.1 package) through CCP4i [18], [24], [25]. Accessible surface and volume calculations were done using both CNS v1.1, areaimol (CCP4 package) with a solvent probe radius of 1.4 Å, VOIDOO and the Protein-Protein Interaction Server (http://www.biochem.ucl.ac.uk/bsm/PP/server) [18], [20], [26], [27].

## Results and Discussion

### Structure of the liganded AtraPBP1 at pH 6.5

**Figure 2:**
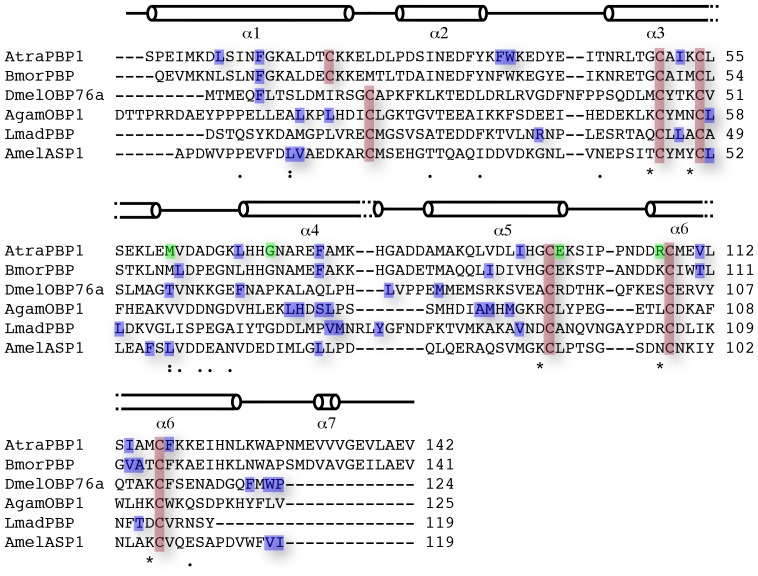
Secondary structure and sequence alignment of some of the structurally characterized pheromone-binding proteins. AtraPBP1 (*Amyelois transitella);* BmorPBP (*Bombyx mori)*; AgamOBP1 (*Anopheles gambiae)*; LUSH/DmelOBP76a (*Drosophila melanogaster*); AmelASP1(*Apis mellifera)*; LmadPBP (*Leucophaea maderae)*. Structurally conserved cysteine residues are boxed in red. Residues involved in hydrophobic contacts with the pheromones (AtraPBP1) are shown in blue. Residues engaged in hydrogen bonding with the ligands are colored in green. Uniprot accession IDs are as follow: D0E9M1 (AtraPBP1); P34174 (BmorPBP); Q8I8T0 (AgamOBP1); O02372 (DmelOBP76a/LUSH); Q9U9J6 (AmelASP1); Q8MTC1 (LmaPBP).

**Figure 3:**
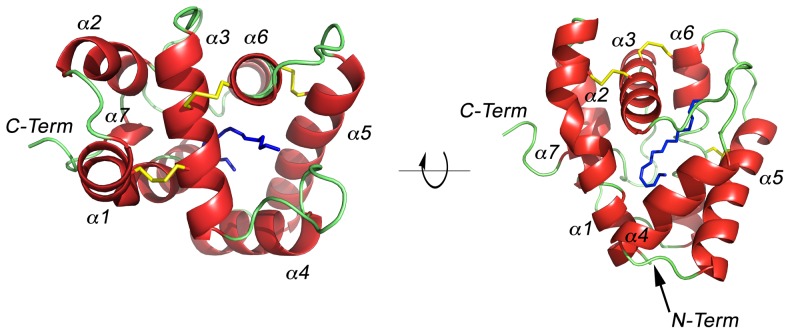
Main-chain trace of AtraPBP1 bound with the major constituent of the, (11*Z*,13*Z*)-hexadecadienal (*Z*11*Z*13-16Ald) with α-helices colored in red, coils in green and disulfide bonds in yellow linking the α-helices α1 to α3 (Cys19-Cys54), α3 to α6 (Cys50-Cys108), α5 to α6 (Cys97-Cys117). The α-helices are sequentially labelled.

**Figure 4:**
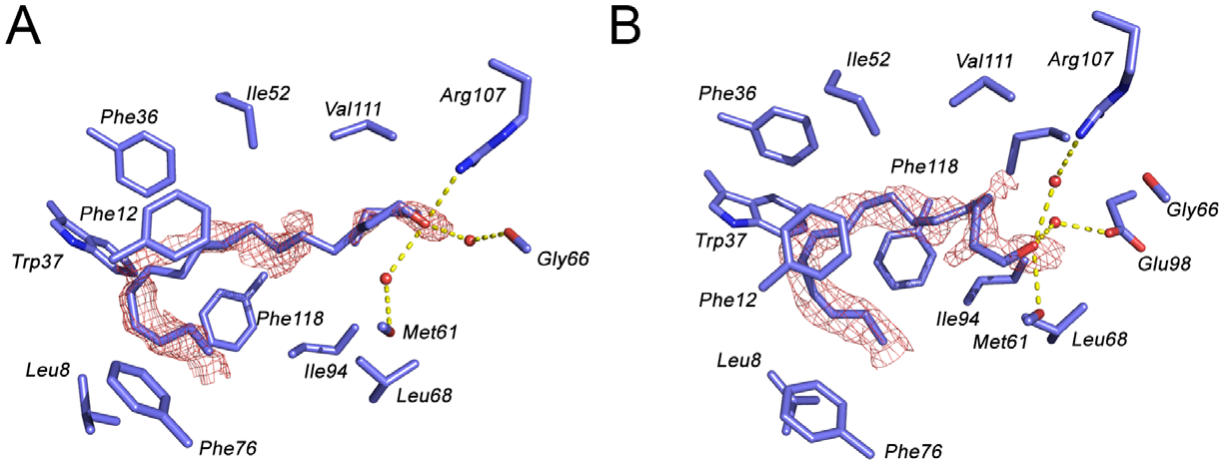
Binding site details: F_o_-F_c_ omit electron density maps contoured at 2σ with (11*Z*,13*Z*)-hexadecadienal (A) and (11*Z*,13*Z*)-hexadecadienol (B) Water molecules involved in hydrogen bonds with the pheromones are displayed as red spheres.

**Figure 5:**
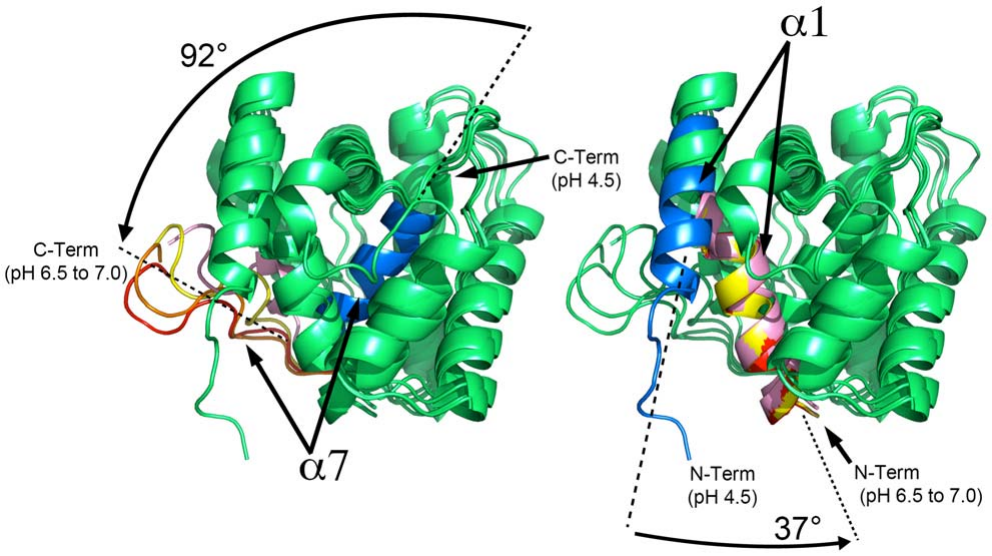
Overview of the pH-dependent movements for the α-helices α1 and α7. Structural overlay between the NMR structure at pH 4.5, the x-ray structure at pH 6.5 and the MD simulations to model the modifications at pH 7.0. Color code: blue (NMR structure at pH 4.5); light pink (x-ray structure at pH 6.5); yellow (pH 7.0 MD at t = 5 ps); orange (pH 7.0 MD at t = 10 ps); red (pH 7.0 MD at t = 12.5 ps). The pH-induced rotations observed in helices 1 and 7 are shown.

**Figure 6:**
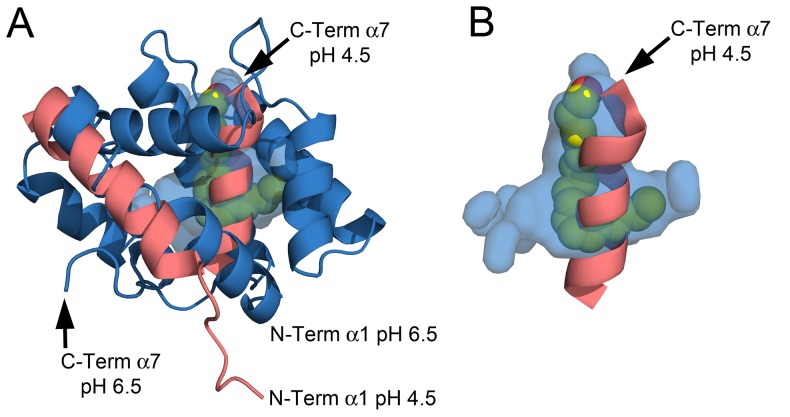
The binding cavity. A) Structural overlay between the NMR structure at pH 4.5 (light red) and the x-ray structure at pH 6.5 (blue). The surface cavity at pH 6.5 is colored in blue and (11*Z*,13*Z*)-hexadecadienal is displayed in space-filling representation. B) De-cluttered detail of the binding cavity at pH 6.5 and the C-term α7 occupying the space at pH 4.5.

The pH 6.5 AtraPBP1 structure complexed with either *Z*11*Z*13-16OH or *Z*11*Z*13-16Ald adopts the same core canonical fold described previously for pheromone-binding proteins consisting of six α-helices connected by loops (Fig. 2, 3). The backbones of the structures with the two different ligands are nearly identical as there is an r.m.s.d. of <1 Å between α-carbons. Only the C-terminus loop (Val135 to Glu137) is divergent. Among the structures of all odorant-binding proteins previously characterized, the pheromone-binding protein from *Bombyx mori* (PDB: 1DQE) is the closest structural neighbor of AtraPBP1, sharing 67% sequence identity and a 1.1 Å r.m.s.d. between equivalent α-carbons (Table 2). Odorant-binding proteins share a sequence identity as low as 12% while maintaining a high degree of structural conservation between them (Table 2) [28]. The AtraPBP1 scaffold contains three-conserved disulfide bonds linking the α-helices α1 and α3 (Cys19-Cys54); α3 and α6 (Cys50-Cys108), α5 and α6 (Cys97-Cys117), which encapsulate the hydrophobic pheromones in the binding site (Fig. 3). The average B-factor for both structures is relatively low with 18.2 Å^2^ (holo *Z*11*Z*13-16Ald) and 13.0 Å^2^ (holo *Z*11*Z*13-16OH) respectively. At 156 Å^3^, the hydrophobic binding cavity in AtraPBP1 is large when compared to other odorant-binding proteins (Table 2) and is lined by a set of non-conserved residues (Fig. 2), which are responsible for conferring specificity. Early in the refinement process, unambiguous 2*F*_o_-*F*_c_ electron densities in the binding pocket were present and therefore were used to model both pheromones in the latter stages (Fig. 4). As with most odorants, these pheromones are highly hydrophobic compounds and one function of PBPs is to carry such molecules across the aqueous sensillar lymph [29]. Accordingly, they are both almost completely sequestered from the solvent by the protein (2.7% and 2.2% solvent accessibility for the alcohol and aldehyde forms respectively) (Fig. 5 and Fig. 6).

**Table 2:** Structural neighborhood of AtraPBP1.

*Source*	*r.m.s.d. (Å)*	*% identity*	*residues*	*Binding cavity vol. (Å^3^)*	*PDB ID*	*reference*
silkworm moth, *Bombyx mori *pheromone-binding protein (BmorPBP)	1.1	67	137	171	1DQE	[16]
malaria mosquito, *Anopheles gambiae *odorant-binding protein 1 monomer (AgamOBP1)	3.0	18	123	27	2ERB	[7]
cockroach, *Leucophaea maderae *pheromone-binding protein (LmadPBP)	3.1	12	117	85	1ORG	[33]
honey bee, *Apis mellifera* antennal-specific**protein 2 (AmelASP2)	2.9	12	123	157	1TUJ	[34]
honey bee, *Apis mellifera* antennal-specific**protein 1 (AmelASP1)	2.8	15	117	128	3BJH	[35]
fruit fly, *Drosophilia melanogaster *odorant-binding protein 76a (LUSH/DmelOBP76a)	2.8	14	124	108	1OOH	[31]

Some OBPs are known to dimerize. In the cases of the mosquito proteins OBP1 from *Anopheles gambiae* and OBP1 from *Culex quinquefasciatus* this is functionally relevant because the dimerization forms a long ∼30 Å tunnel that traverses the interface in which ligands are bound [7], [30]. Structures from crystals of the honeybee ASP1 grown at high pH indicate a domain-swapped dimer at high pH [6]. NMR experiments have suggested that AtraPBP1 also dimerizes in solution in response to a pH increase from 4.5 to 7.0 [8], [13]. The structure from crystals grown at pH 6.5 shows no evidence of this as there are no crystallographic or noncrystallographic 2-folds which would be necessary for a symmetrical dimer. Moreover, buried surface areas corresponding to lattice contacts in AtraPBP1 range from 300–500 Å^2^ each as opposed to approximately 1200 Å^2^ to 1350 Å^2^ found in the dimeric interfaces seen in AgamOBP1, LUSH/DmelOBP76a and ASP1 (Table 2) [6], [7], [31].

### Binding of the aldehyde and alcohol pheromone constituents

The overall hook conformation of the hydrocarbon chain in both pheromone constituents is largely dictated by the *cis* desaturations at positions 11 and 13 (Fig. 1). The binding site is highly complementary to these linear molecules in this particular conformation and is unlikely to be capable of binding molecules, which are longer or significantly diverge in shape. Both pheromones are firmly stabilized into the binding pocket primarily by an array of hydrophobic interactions. In addition, a specific set of hydrogen bonds for the small polar head region of each pheromone complete the anchoring of both molecules. The hydrophobic interactions are mediated by the side-chains of Leu8, Ile52, Met61, Leu68, Ileu94, Val111 and Ile114 that make numerous interactions with the carbon chain. In addition, a stacked arrangement of a quartet of phenylalanines at positions 12, 36, 76 and 118 interact with the ligands near the desaturated carbons (Fig. 4).

In the case of the *Z*11*Z*13-16Ald pheromone constituent, the oxygen of the aldehyde group is hydrogen-bonded to the positively charged Arg107 guanidinium group (Fig. 4A). In addition, two water-mediated hydrogen bonds with the backbone oxygen of both Met61 and Gly66 complete the binding mesh of *Z*11*Z*13-16Ald in AtraPBP1. Possible x-ray radiation-induced damage was found in the *Z*11*Z*13-16Ald bound structure at the early stages of the refinement consisting of an unambiguous, discontinuous 2F_o_-F_c_ electron density at 1.14 Å resolution located where the disulfide bond between Cys19 and Cys54 should be. The same bond was found intact on the refined structure from a preliminary dataset collected on a home source at 1.54 Å using the same crystal (data not shown).

The alcoholic component, *Z*11*Z*13-16OH binds in a manner similar to *Z*11*Z*13-16Ald (Figs. 2 and 4) and shares the same array of hydrophobic interactions as *Z*11*Z*13-16Ald. The differences appear in the stabilization of the polar region of the ligand which accounts for only ∼5% of the molecular surface of each compound. The oxygen of *Z*11*Z*13-16OH functions as a hydrogen bond donor to the backbone oxygen of Met61. Two water-mediated hydrogen bonds between a guanidinium nitrogen of the Arg107 side-chain and a hydroxyl group of Glu98 complete the binding network. This set of residues is not conserved with other homologous pheromone-binding proteins and this variability may play a key role in the binding selectivity and specificity of pheromones in other PBPs (Fig. 2). The major difference between the modes of binding of *Z*11*Z*13-16OH and *Z*11*Z*13-16Ald involve the reshuffling of these three hydrogen bonds mediating the polar contacts with each ligand.

### Comparison between the NMR apo-structure at pH 4.5 and the x-ray holo-structures at pH 6.5: implications for the pH-dependent extrusion of C-terminal helix

Many odorant-binding proteins that have been studied are believed to load and release pheromones through a pH-dependent conformational rearrangement. This effect has first been described for BmorPBP, the PBP from the silkworm moth *Bombyx mori* where the C-terminal region is unstructured in the pheromone-bound PBP at basic pH [16]. However at acidic pH, the C-terminal region forms an α-helix that competes with the pheromone by inserting into the binding pocket [9], [32]. In a previous study, Xu et al. determined the apo-AtraPBP1 NMR structure at pH 4.5 and demonstrated that the homologous C-terminal region of AtraPBP1 plays a key role by forming a helical structure at pH 4.5 which occupies the pheromone binding pocket [13]. At pH 7.0, the deprotonation of His80 and His95 disrupts the salt bridges anchoring the helix α7 in the hydrophobic cavity, which trigger the movement of the helix outward from the binding pocket, enabling a path for the binding of pheromones [8]. Removal of the entire C-terminal α7 helix along with half of the α6 helix (residues 129–142) causes more than 100-fold increase in pheromone binding affinity at pH 5 and only a 1.5-fold increase at pH 7 [8]. Mutation of the histidines responsible for anchoring α7 yields protein, which is capable of binding at low pH. Taken together, these studies suggest that the pH-dependent extrusion of the C-terminal helix α7 from pH 4.5 to 7.0 contribute to expose a hydrophobic cavity for the binding of pheromone.

In the present study, pheromone-bound crystal structures of AtraPBP1 at high pH bring complementary structural insights to the pH-dependent release mechanism for pheromones. At pH 6.5, the C-terminal helix α7 is partially disordered and extruded to accommodate the pheromone, which is consistent with the previous studies of AtraPBP1 [8], [13]. The structures in complex with both the alcohol and aldehyde demonstrate that the pheromone is almost completely inaccessible to the solvent (Figs. 5 and 6). Similarly, most of the other structurally characterized PBPs either substantially or completely sequester ligands from the solvent. A significant conformational change associated with the N-terminal helix α1 is evident when structures determined at high and low pH are compared. This helix is rotated approximately 37° toward the binding pocket and it contributes to the formation of the ligand binding site and sequestration of the ligand (Fig. 5 and Fig. 6A). Indeed, two important pheromone interacting residues, Leu8 and Phe12 are brought into proper binding position by the reorientation of this helix.

**Figure 7:**
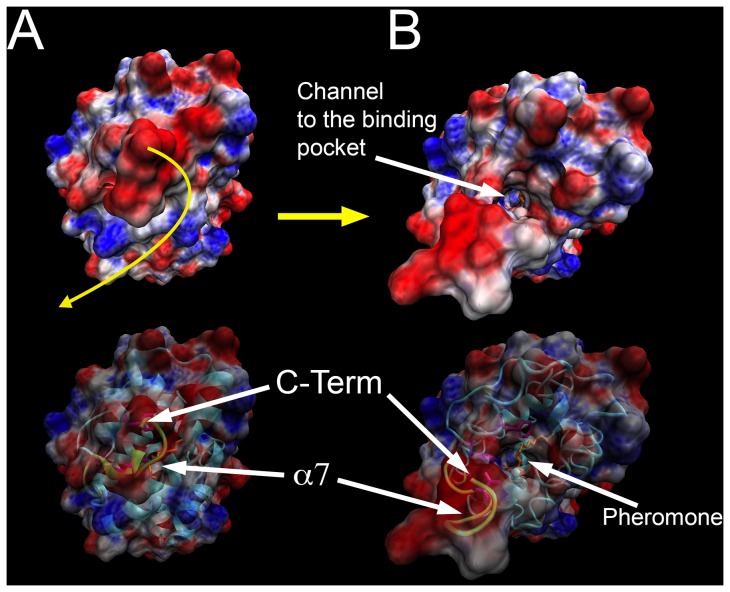
Molecular dynamics revealing the channel to the binding pocket at pH 7.0 along with the electrostatic surface properties. A) MD at t = 0 ps; B) MD at t = 12.5 ps. The motion of the helix α7 is displayed in yellow. Electrostatic calculations are represented by blue for positive charge and red for negative charge with unit +5/−5 kT/e. Electrostatic calculations were done with APBS V1.2.1 and rendered with both VMD V1.8.7 and PyMOL V1.2 [21], [22].

Taken together, the analysis of the structures at pH 4.5 and 6.5 suggests a mechanism for ligand binding and dissociation. At acidic pH and in the absence of pheromone, the α7 helix rotates inside the hydrophobic binding pocket and is anchored by a pair of salt bridges (His80-Glu132, His95-Glu141). As the pH is increased from 4.5 to 7.0, the deprotonation of both His80 and His95 disrupts these interactions, enabling helix α7 to move outside of the binding cavity. Subsequently, helix α7 becomes disordered which contributes to opening a narrow path to the hydrophobic binding cavity, which is inadequate for the transit of the ligand (Fig. 7). Meanwhile, the N-terminal α1 helix rotates ∼37° towards the entrance to the binding pocket to aid in shutting the path at pH 7.0 when a pheromone is bound (Fig. 5 and Fig. 6). The N-terminal helix α1 rotates in synergy with the extrusion of the C-terminal helix α7 during the pH transition from acidic to neutral.

The mechanism by which AtraPBP1 load and unload pheromones involves synergistic movements of both helices α1 and α7 triggered while transitioning through a pH gradient. In addition, the protein's specificity for particular shapes and sizes of molecules may imply the existence of a filter which could be located at the entrance of the narrow tunnel leading to the inner binding cavity. Both the NMR structure at pH 4.5 and the x-ray structures at pH 6.5 are snapshots of the conformation of AtraPBP1 during the pH-induced structural transition. At pH 4.5, AtraPBP1 is devoid of ligand with the α7 helix occupying the inner binding cavity in a shut conformation inaccessible for the solvent. The transition from pH 4.5 to 6.5 results in significant conformational modifications with the synergetic movement of helices α1 and α7. However, the binding cavity at pH 6.5 is isolated from the solvent. Our data indicate that both *Z*11*Z*13-16OH and *Z*11*Z*13-16Ald are virtually solvent inaccessible. Taken together, it clearly appears that the pH transition above pH 6.5 results in the opening of a narrow tunnel to the inner cavity. The question of whether the extrusion of the α7 helix is solely responsible for opening a path to the binding pocket cannot be answered using the currently available NMR and x-ray structures alone. Despite many attempts, crystals of AtraPBP1 could not be grown above pH 6.5, which would have given decisive answers on the motion of both N- and C-terminal regions along with the structural properties of the gating system to the binding pocket. NMR studies reported an extended and disordered N-terminal region above pH 6.5, which is likely to hinder any crystal growth at neutral pH [8], [13].

In order to understand the motions necessary to access the binding cavity, we used molecular dynamics (MD) to model the motions of the helices α1 and α7 that would occur in neutral pH environment. After a 12.5 ps (12,500 steps) MD simulation using the 1.1 Å refined crystal structure as a starting model, the helix α7 becomes fully disordered and rotates away exposing the long narrow channel to the hydrophobic pocket. Meanwhile, the α1 helix has a less significant movement and does not contribute to the channel opening. This agrees with the recent NMR analysis of AtraPBP1 at pH 7.0 [8], [13]. These modeling experiments are consistent with the idea that the pH transition from 4.5 to 7.0 leads to a ∼37° rotation of the N-terminus α1 helix in order to accommodate the large ∼92° rotation of the C-terminus α7 helix from the inside pocket to the outward extended and disordered conformation (Figs. 6 and 7). Analysis of the MD simulations at 12.5 ps indicates that the Leu16, Phe30, Trp37, Phe73, Val134 sidechains which extend around the mouth of the narrow tunnel, appear to form a tight hydrophobic filter (Fig. 7). This set of residues may act as a selectivity filter and perhaps facilitate the motion of the pheromones through the channel.

### Concluding remarks

In this study, we solved the high resolution structures of the pheromone-binding protein 1 from *A. transitella* (AtraPBP1) complexed with *Z*11*Z*13-16OH and *Z*11*Z*13-16Ald constituents of *A. transitella* sex pheromones. Previous studies have reported the NMR structure of the non-liganded structure of AtraPBP1 in acidic conditions and highlighted the key role of the C-terminal region in controlling the pheromone binding through the extrusion of the C-terminal helix α7. The structural comparison of AtraPBP1 liganded structures at both pH 4.5 and pH 6.5 leads to the proposal that a novel rotation of the N-terminal helix α1 operates in synergy with the extrusion of the C-terminal helix α7 during the pH transition from acidic to neutral. It causes a narrow path to open to the binding pocket, which may restrict the size of ligands that are able to access the binding pocket.
